# Analysis of a Preliminary microRNA Expression Signature in a Human Telangiectatic Osteogenic Sarcoma Cancer Cell Line

**DOI:** 10.3390/ijms22031163

**Published:** 2021-01-25

**Authors:** Gaia Palmini, Cecilia Romagnoli, Simone Donati, Roberto Zonefrati, Gianna Galli, Francesca Marini, Teresa Iantomasi, Alessandra Aldinucci, Gigliola Leoncini, Alessandro Franchi, Giovanni Beltrami, Domenico Andrea Campanacci, Rodolfo Capanna, Maria Luisa Brandi

**Affiliations:** 1 Department of Experimental and Clinical Biomedical Sciences, University of Florence, 50134 Florence, Italy; gaia.palmini@unifi.it (G.P.); cecilia.romagnoli@unifi.it (C.R.); simone.donati@unifi.it (S.D.); roberto.zonefrati@unifi.it (R.Z.); gianna.galli@unifi.it (G.G.); francesca.marini@unifi.it (F.M.); teresa.iantomasi@unifi.it (T.I.); gigliola.leoncini@unifi.it (G.L.); 2 Central Laboratory, Azienda Ospedaliero-Universitaria Careggi, 50134 Florence, Italy; aldinuccia@aou-careggi.toscana.it; 3 Department of Translational Research and of New Technologies in Medicine and Surgery, University of Pisa, 56126 Pisa, Italy; alessandro.franchi@unipi.it (A.F.); rodolfo.capanna@unipi.it (R.C.); 4 Ortopedia Oncologica Pediatrica, AOU Careggi-AOU Meyer, 50139 Florence, Italy; beltramig@aou-careggi.toscana.it; 5 SOD Ortopedia Oncologica e Ricostruttiva, AOU Careggi, 50134 Florence, Italy; domenicoandrea.campanacci@unifi.it; 6 Fondazione Italiana Ricerca sulle Malattie dell’Osso (FIRMO Onlus), 50141 Florence, Italy

**Keywords:** microRNAs, cancer stem cells, telangiectatic osteosarcoma

## Abstract

Telangiectatic osteosarcoma (TOS) is an aggressive variant of osteosarcoma (OS) with distinctive radiographic, gross, microscopic features, and prognostic implications. Despite several studies on OS, we are still far from understanding the molecular mechanisms of TOS. In recent years, many studies have demonstrated not only that microRNAs (miRNAs) are involved in OS tumorigenesis, development, and metastasis, but also that the presence in high-grade types of OS of cancer stem cells (CSCs) plays an important role in tumor progression. Despite these findings, nothing has been described previously about the expression of miRNAs and the presence of CSCs in human TOS. Therefore, we have isolated/characterized a putative CSC cell line from human TOS (TOS-CSCs) and evaluated the expression levels of several miRNAs in TOS-CSCs using real-time quantitative assays. We show, for the first time, the existence of CSCs in human TOS, highlighting the in vitro establishment of this unique stabilized cell line and an identification of a preliminary expression of the miRNA profile, characteristic of TOS-CSCs. These findings represent an important step in the study of the biology of one of the most aggressive variants of OS and the role of miRNAs in TOS-CSC behavior.

## 1. Introduction

Osteosarcoma (OS) is the most common primary skeletal malignancy in children and young adults, accounting for 20 to 35% of all malignant primary bone tumors [1,2]. OS mainly arises at the metaphyseal level of the long bones; in particular, the distal femur, proximal humerus, and proximal tibia [3,4,5]. Various high-grade subtypes of OS have been described in relation to the histology and the area of interest of the primary tumor bulk. Among these is telangiectatic osteogenic sarcoma (TOS) [6,7]. TOS is an aggressive variant of OS with an incidence of 3 to 6% of all diagnosed high-grade OS [7,8].

TOS is characterized by distinctive radiographic, macroscopic, and microscopic features and by different prognostic implications. The most common location at TOS presentation is the metaphyses of long bones and, in particular, the proximal tibia (16.9%) and distal femur (41.6%) [9,10,11,12]. However, TOS can also occur in atypical locations for OS, such as craniofacial bones, jaw, mandible, vertebrae, and soft tissues [13,14,15,16,17]. TOS of the soft tissues is a rare extraskeletal OS, characterized by the production of osteoid matrix at soft tissue levels, without any attachment to bone or periosteum. This rare TOS variant usually affects adults older than 50 years of age [18,19,20].

TOS was described for the first time in 1853 by Paget [21]. At first it was not considered a bone tumor, but rather an osseous cystic aneurysm, since the affected bone presented a lesion with hemorrhagic and cystic changes [22].

In 1922, Ewing [23] was the first to clarify and classify this particular bone lesion as an aggressive variant of osteogenic sarcoma. In 1976, in relation to the difficulty of TOS diagnosis, Matsuno et al. proposed three diagnostic criteria [8], which are still used. These criteria are: 1. Radiographically, the lesion observed shows lytic and destructive appearance without an evaluable sclerosis, 2. Macroscopically, the lesion appears as a cavity filled with little neoplastic solid tissue, but always without sclerotic areas; 3. Microscopically, the cancerous tissue presents single or multiple cavities, which are filled with blood or necrotic tumor areas. Later, ultrastructure analysis of TOS samples using electron microscopy showed that the cystic cavities are filled with clotted blood and segmented by several septa, which often contain tumor cells. Malignant cells (anaplastic, osteoblast- and fibroblast-like tumor cells) can produce the osteoid matrix, which appears as a lacelike material between the observed septa and the tumor cells [24]. Electron microscopy has also shown that certain areas of TOS present the typical pattern of OS lesions, where osteoblast-like tumor cells are embedded in a large amount of osteoid matrix [24].

The management of all diagnosed TOS of the extremities is first characterized by surgical excision of the primary tumor bulk by amputation, or limb-salvage surgery, when possible. In addition to surgery, adjuvant preoperative chemotherapy plays an important role in the treatment of TOS, in particular in TOS patients who present metastases at diagnosis. Several studies have reported that TOS responds well to treatment compared to OS, and this is most likely due to the “non-solid” aspect of the bone lesion [25,26]. Nevertheless, the survival rate at five years is poor because of local recurrences or distant metastases (i.e., lung metastasis), which are very common in case of the presence of metastases at diagnosis [27,28]. At the same time, all case reports regarding extraosseous TOS of soft tissues and TOS of unusual locations (i.e., jaw, mandible, spine or skull) present poor prognosis given that, in these cases, surgery is often impossible, and the patients present recurrences or lung metastases [12,13,14,15,16,17,18,19].

MicroRNAs (miRNAs) are non-coding, small in length RNAs (18–24 nucleotides), with the particular ability to regulate several genes at the post-transcriptional level, having the potential to regulate several biological processes, such as the differentiation, progression, apoptosis and proliferation of tumor cells [29]. In 2002, Calin et al. described for the first time that miRNA dysregulation could translate into the manifestation of lymphocytic leukemia [30]. Since then, several studies have reported that miRNAs could be involved in the tumorigenesis of several tumors [31,32]. Recent studies have suggested miRNA implication in skeletal tissue development, and that an aberrant expression of miRNAs could be involved in bone sarcoma development and progression, resulting in a tumor miRNA signature, which could be useful for the classification, diagnosis, and evaluation of the response to therapeutic treatments in bone sarcomas [33,34].

In recent decades, several studies have begun to investigate the role of various miRNAs in OS to try to find new molecular diagnostic, prognostic and therapeutic targets, with the final aim of understanding the biology of OS, and to find new and more effective molecular therapies against this aggressive primary bone tumor.

Another important scientific finding related to understanding the biology of bone tumors has been the demonstration of the presence in both liquid and solid tumors, and in OS of a subpopulation, of a specific type of neoplastic cells, called cancer stem cells (CSCs) [35,36,37,38]. CSCs have been described as the tumor cellular population responsible for maintaining the primary tumor bulk, recurrences and metastases, even after conventional multidisciplinary adjuvant and neoadjuvant chemotherapy [39,40,41].

Despite the recent findings regarding OS, nothing related to the miRNA expression profile and the presence of CSCs has been described in TOS.

Therefore, the current study was first designed to investigate the presence of CSCs in one of the most aggressive and malignant forms of OS, establishing an in vitro model consisting of cells that can be considered as a putative TOS-CSCs line.

The second aim of this study was to evaluate a characteristic miRNA expression profile of TOS-CSCs, to understand the role of miRNA in TOS biology and find a miRNA profile, which could represent an important diagnostic tool for predicting tumor sensitivity to treatment, and for identifying new molecular targets to development new and more effective therapies against TOS.

## 2. Results

### 2.1. Establishment of a Putative TOS-CSCs Cell Line

**Figure 1:**
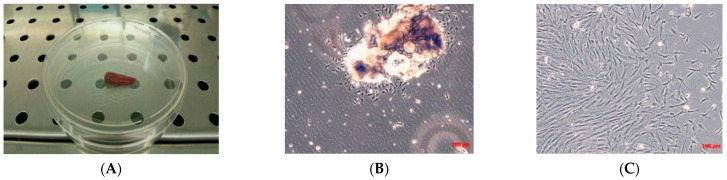
Biopsy specimen and primary cell culture of TOS. (**A**) Biopsy specimen of TOS by needle aspiration. Observation in phase contrast of primary cell culture of TOS after 24 h from enzymatical and mechanical treatment of the biopsy (**B**) and after three days (**C**). Original magnification 10×.

Telangiectatic osteosarcoma (TOS) sample, obtained by surgical resection of a part of the tumor (Figure 1A), permits the isolation of only one osteosarcoma cell line if treated precisely, as described in the materials and methods section.

The number of cells isolated from the osseous bioptic fragment was very low, and for the primary TOS cell line one month was necessary to reach confluence in a 100 mm tissue dish. After one month, a primary cell line of TOS was obtained and marked as TOS1 (Figure 1B,C).

**Figure 2:**
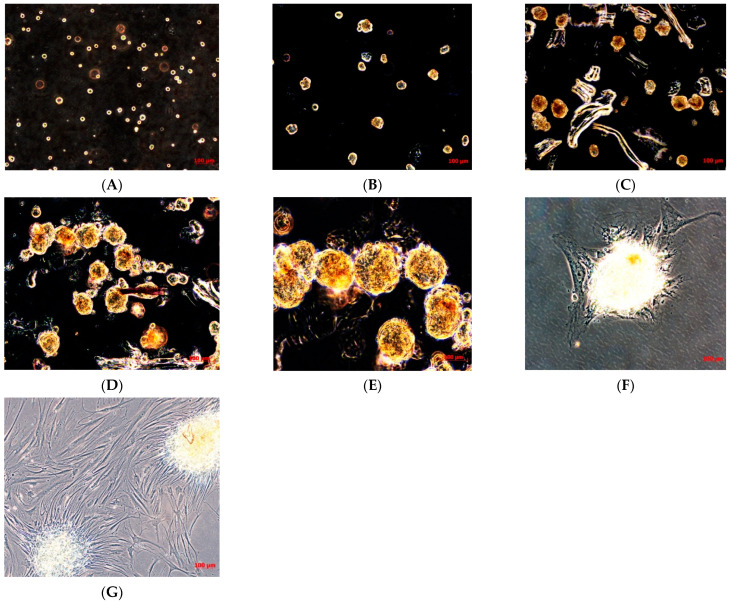
Sarcosphere formation assay on TOS and sarcosphere adhesion. Sarcosphere formation under non-adherent conditions in TOS1 cell line after 24 h (**A**), 7 days (**B**), 14 days (**C**), and 21 days (**D**,**E**). Sarcospheres from TOS1 cell line showed adherent expansion by reintroduction and reculturing in monolayer, adherent conditions at 48 h from the isolation (**F**) and after 7 days (**G**). Observation in phase contrast. Original magnification 10×-20-40×.

Afterwards, when the cells had grown, they were subcultured to obtain a sufficient number of cells to cryopreserve the primary cell line obtained, and to isolate the cancer stem cells (CSCs). When the TOS1 cell line was at the second passage of subculture and reached the confluence, cells were detached and plated in 6-well-ultralow attachment plates specific for the sarcosphere assay. These plates permit the generation of a stressful condition, preventing the attachment of cells to the substrate, completely inhibiting the anchorage-dependent processes, which can be sustained only by cancer stem cells. Twenty-four hours after the sarcosphere assay, cells appeared floating and isolated from one another (Figure 2A). After seven days, small spherical colonies called “sarcospheres” could be observed (Figure 2B). We observed that sarcospheres grew over time (Figure 2C,D) until 21 days, when we stopped the assay, proceeding to isolate all the sarcospheres formed from each well of the plate.

The sarcospheres, as described in the Materials and Methods Section, were placed immediately in normal attachment 60 mm diameter tissue culture dishes, under normal adherent conditions in GM. Forty-eight hours after the placement in adherent conditions, the colonies’ attachment and their adherent expansion over the time was observed (Figure 2F,G).

Hence, after isolation, the isolated cell line obtained, consisting of cells that can be considered as putative CSCs, was marked as TOS1-CSCs line. It had to be grown and subcultured to cryopreserve this unique cell line and to obtain enough cells to perform the analyses/assays to characterize the cancer stem phenotype typical of CSCs.

### 2.2. In Vitro Characterization of the Isolated Cellular Model

**Figure 3:**
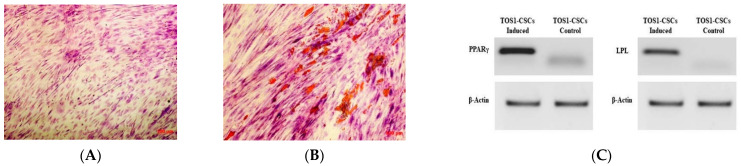
Adipogenic differentiation assay and expression of adipogenic gene markers. Adipogenic differentiation at 0 days (**A**) and after 14 days (**B**) of induction by cytochemical staining with OIL Red O. Red shows the lipidic vesicles, violet shows the nuclei counterstained by hematoxylin. Observation is in brightfield. Original magnifications: 10×. (**C**) RT-PCR show the expression of PPARγ (left) and of LPL (right) in TOS1-CSCs after 14 days of adipogenic induction.

Adipogenic differentiation was not observed in the TOS-CSC line at 0 days, while after 14 days of adipogenic induction, some cells showed the presence of intracellular vesicles containing drops of liquid of variable shape and size (Figure 3A,B).

The adipogenic differentiation for the isolated cell line was also confirmed by RT-PCR of adipocyte specific marker genes (i.e., LPL and PPARγ), which are involved in the adipogenesis process. In absence of adipogenic differentiation, qualitative RT-PCR showed the lack expression of both these genes, which can be revealed by RT-PCR after 14 days of differentiation (Figure 3C).

**Figure 4:**
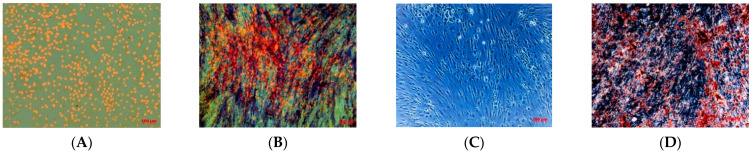
Osteogenic differentiation assay_ALP and HA. Osteogenic differentiation_ALP at 0 days (**A**) and after 10 days (**B**) of induction by cytochemical staining for ALP with Fast Blue BB. Blue shows the ALP + cells; in orange/red the nucleus counterstained by Propidium Iodide. Composite observation in brightfield and fluorescence. Osteogenic differentiation_HA at 0 days (**C**) and after 20 days (**D**) of induction by cytochemical staining for Hydroxyapatite (HA) with Alizarin Red S. The cells only are contrasted in blue/grey, and the grainy deposits of HA are stained in red. Observation in phase contrast. Original magnification: 20×.

After, we evaluated the osteogenic differentiation capacity of isolated cell line. After only 10 days of induction, TOS1-CSCs showed approximately 85% of the cellular population positive to presence of ALP (Figure 4A,B) and showed several calcium mineralized deposits after 20 days of induction (Figure 4C,D).

**Figure 5:**
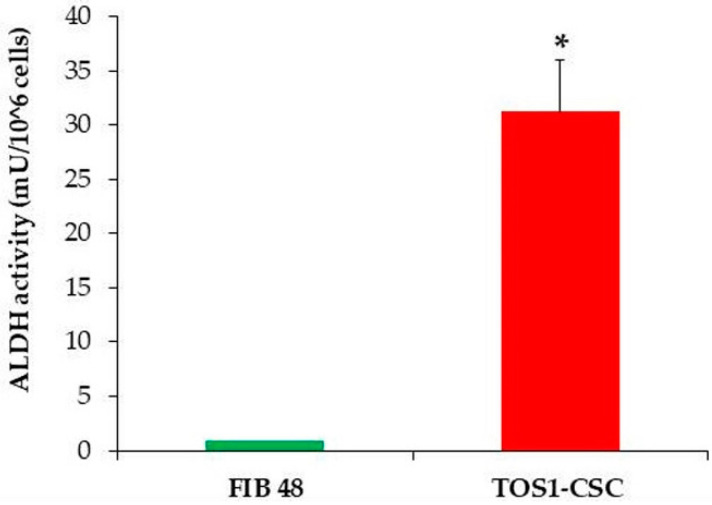
ALDH activity assay. The ALDH colorimetric assay detected high levels of ALDH activity in TOS1-CSCs cell line. However, the assay detected the absence of this activity in the finite differentiated cell line of fibroblasts, FIB48. Error bars: SD. *: *p* < 0.05 vs. FIB48.

We have also reported a good rate of clonogenic efficiency (33%) of TOS1-CSCs. In relation to several studies that have shown that high levels of ALDH activity are characteristic of CSCs, we evaluated ALDH activity. The assay has shown that TOS1-CSCs present high levels of ALDH activity vs. fibroblast cell line, which was used as negative control of this activity (Figure 5).

Soft agar assay showed the capacity of the TOS1-CSCs cell line to grow in soft agar and to form large spherical colonies.

**Figure 6:**
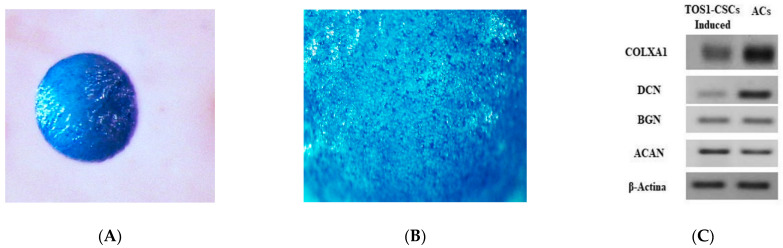
Chondrogenic differentiation assay and expression of hyaline-chondrogenic gene markers. Histological evaluation of TOS1-CSC condrogenic 3Dimensional cell pellet at 21 days (**A**). Alcian Blue stain demonstrates positive glycosaminoglycan production (**B**). Stereomicroscopy images. Original magnification: 16× and 40×. (**C**) RT-PCR show the expression of COLXA1, DCN, BGN, and ACAN in TOS1-CSCs (left) compared to their expression in a primary cell line of human articular chondrocytes (ACs) (control) (right) after 21 days of chondrogenic induction.

Chondrogenic differentiation was observed after 21 days, observing the formation of a “spherical pellet”, which resulted to be positive to Alcian Blue staining (Figure 6A,B). No spherical pellets have been observed in induced TOS1 cell line.

Alcian Blue staining is a specific immunocytochemical staining used to evaluate the presence of glycoproteins, which are characteristic of hyaline cartilaginous tissue. Cells at 21 days of chondrogenic differentiation show the gene expression of the hyaline chondrogenic gene markers (Type X Alpha Collagen I (COLXA1), Aggrecan (ACAN), Decorin (DCN) and Biglycan (BGN)), which were evaluated by qualitative PCR analysis (Figure 6C).

**Figure 7:**
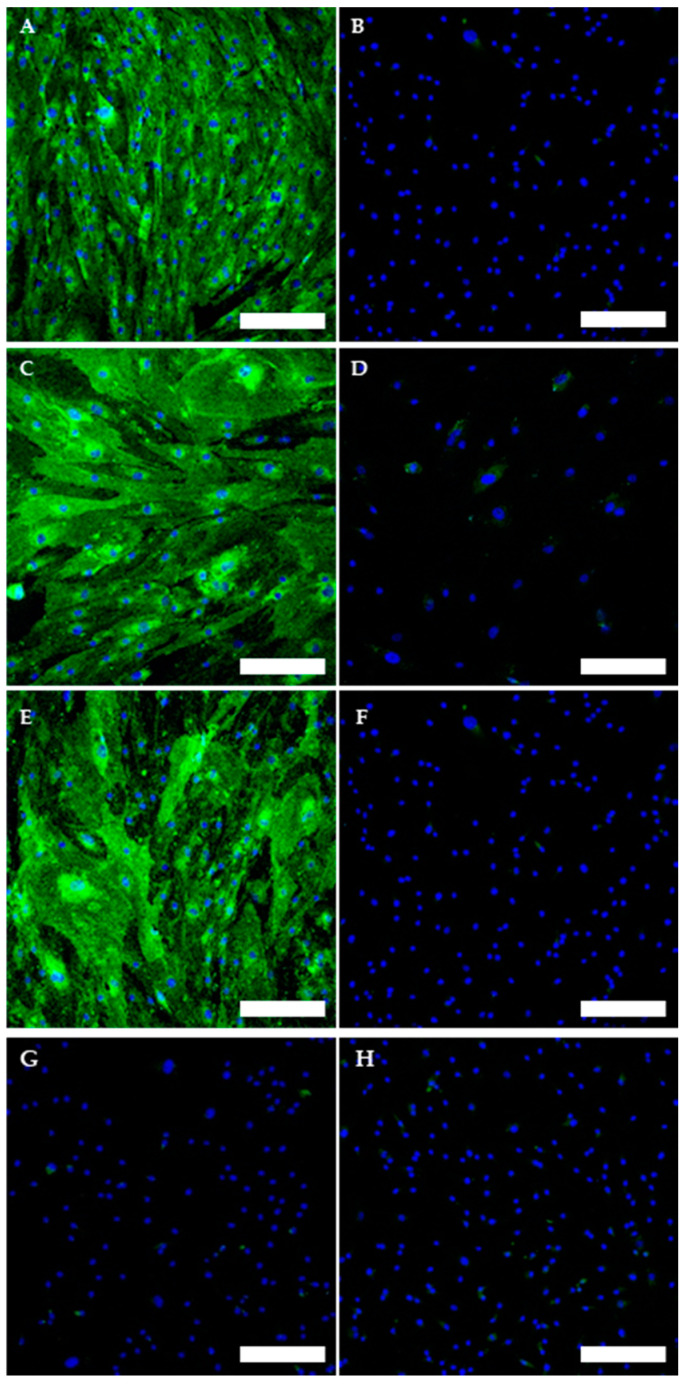
Immunofluorescence staining of Mesenchymal Stem Cell (MSC) and of Hematopoietic surface markers. Immunofluorescence staining of CD44 (**A**), CD105 (**C**), CD90 (**E**) and of CD45 (G) of the TOS1-CSC cell line. Respectively, CD44 (**B**), CD105 (**D**), CD90 (**F**) and of CD45 (**H**) in a human fibroblast cell line (negative control). LSCM in conventional colors: green for MSC markers and blue for nuclei. Original magnification: 10×. Bar size: 100 µm.

The TOS1-CSCs line showed positivity for the surface mesenchymal stem cell (MSC) markers (CD44, CD105 and CD90) (Figure 7A–F). On the contrary, the TOS1-CSCs line resulted to be completely negative for the hematopoietic surface marker CD45 (Figure 7G,H).

**Figure 8:**
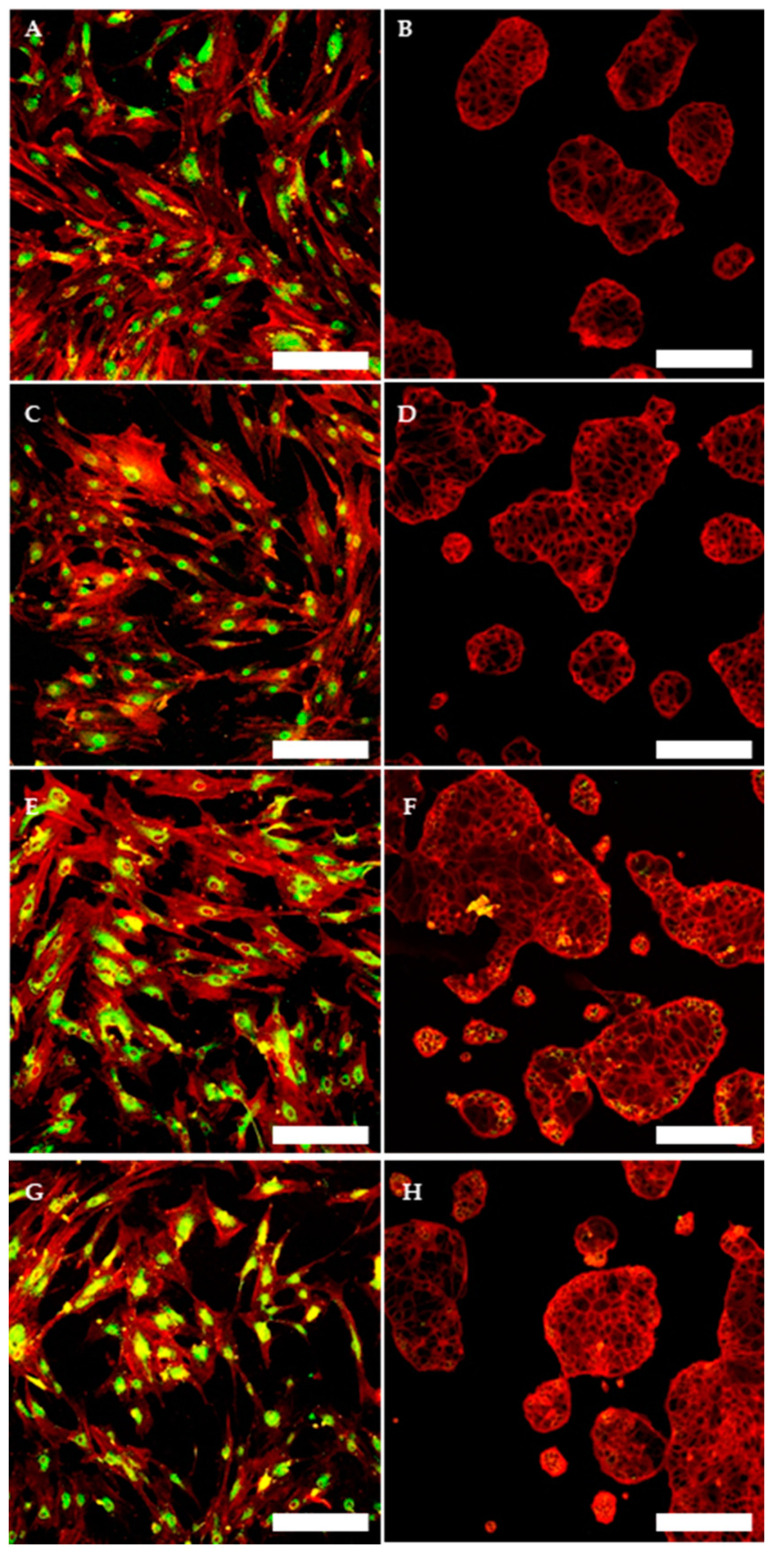
Immunofluorescence staining of embryonic stem cell (ESC) markers. Immunofluorescence staining of Nanog (**A**), of KLF4 (**C**), of Sox2 (**E**) and of POU5F1 (**G**) of TOS1-CSCs line and of HCT8 cell line (**B**,**D**,**F**,**H**) (negative control). LSCM in conventional colors: green for ESC nuclear/perinuclear markers and red for cytoskeleton. Original magnification: 10×.

To evaluate and confirm the MSC phenotype flow, cytometric analyses were performed. One hundred percent of TOS-CSCs resulted to be CD45-; 99.8% of TOS1-CSCs were CD44+/CD90+; 99.4% of TOS1-CSCs were CD105+/CD44+. The isolated cell line also showed positivity for nuclear/perinuclear embryonic stem cell (ESC) markers (Nanog, KLF4, SOX2 and POU5F1) (Figure 8A–H).

**Figure 9:**
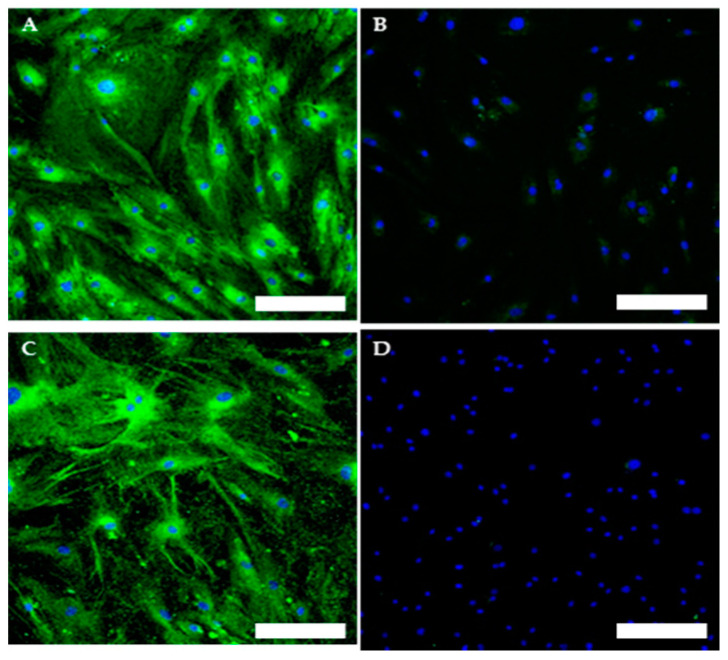
Immunofluorescence staining of neoplastic markers. Immunofluorescence staining of c-Kit (**A**) and of Nestin (**C**) of TOS1-CSC cell line. Immunofluorescence of c-Kit (**B**) and of Nestin (**D**) of human fibroblast cell line (negative control). LSCM in conventional colors: green for c-Kit and Nestin and blue for nuclei. Original magnifications: 10×. Bar size: 100 µm.

Finally, the TOS1-CSCs line showed a high positivity for two neoplastic markers (c-Kit and Nestin) (Figure 9).

Using qualitative RT-PCR analysis, we assessed the expression of the SATB2 gene, which is a marker gene for all the OS genes, in the TOS1 primary cell line and the TOS1-CSC cell line.

**Figure 10:**
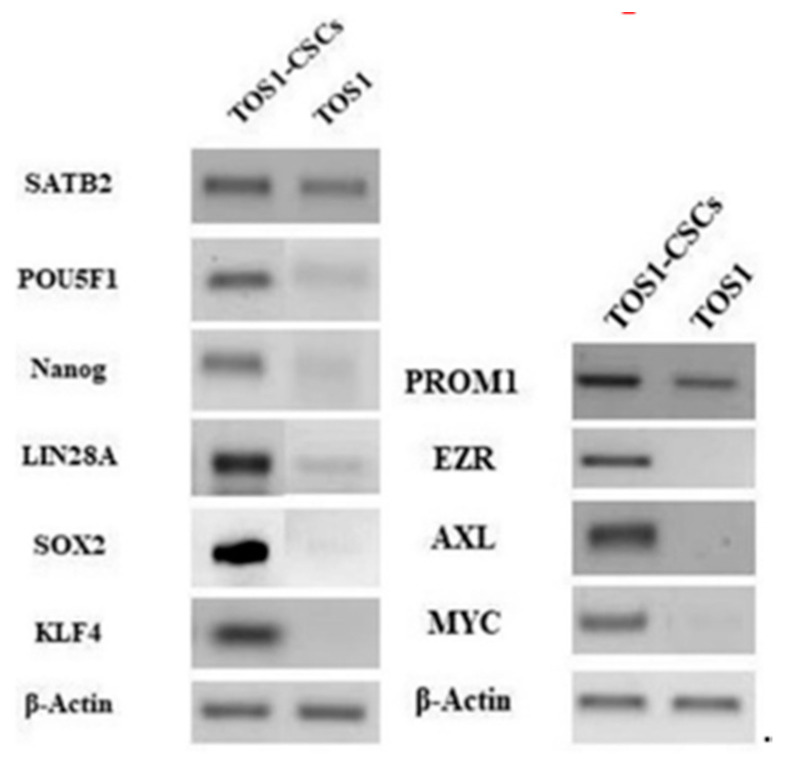
Gene expression in isolated RT-PCR showed the expression of SATB2 and PROM1 gene in TOS1 primary cell culture and in the isolated TOS1-CSC cell line. In addition to this, RT-PCR also showed the expression of the ESC marker genes and of EZR, AXL and MYC only in the TOS1-CSC cell line.

We also noticed the ESC markers (i.e., Nanog, Sox2, KLF4, LIN28A, POU5F1) and that other specific CSCs marker genes (PROM1, EZR, AXL and MYC) were expressed only in the isolated TOS1-CSCs line (Figure 10).

**Figure 11:**
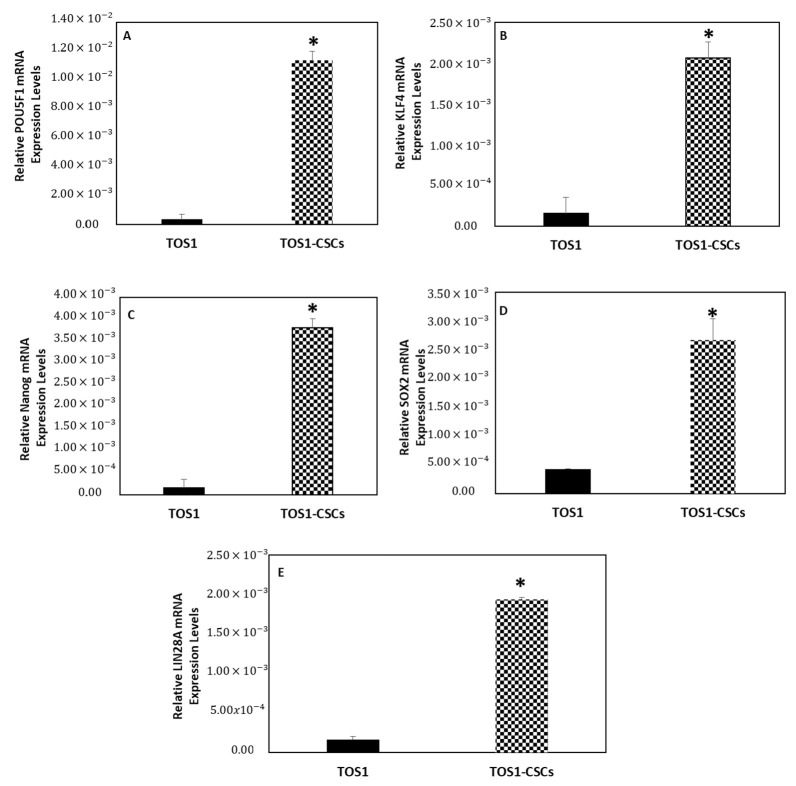
Expression levels of ESC gene markers. The relative POU5F1 (**A**), KLF4 (**B**), Nanog (**C**), SOX2 (**D**) and LIN28A (**E**) mRNA expression levels in TOS1-CSC cell line was determined by RT-qPCR. The results are expressed relative to GAPDH mRNA levels. Data represent the mean with standard deviation (*n* = 3). * *p* < 0.05 versus TOS1 cell line.

After, we also measured the expression levels of the ESC marker genes by quantitative RT-PCR (RT-qPCR). The results showed that KLF4, Nanog, POU5F1, SOX2 and LIN28A mRNA are expressed at very low levels in the primary cell line of TOS, while these are significantly upregulated in the isolated TOS1-CSC cell line (Figure 11A–E).

Once the TOS1-CSCs cell line was characterized, as a putative CSCs line, a preliminary expression miRNA profile was evaluated.

### 2.3. miRNA Expression Profile in TOS-CSCs Model

**Figure 12:**
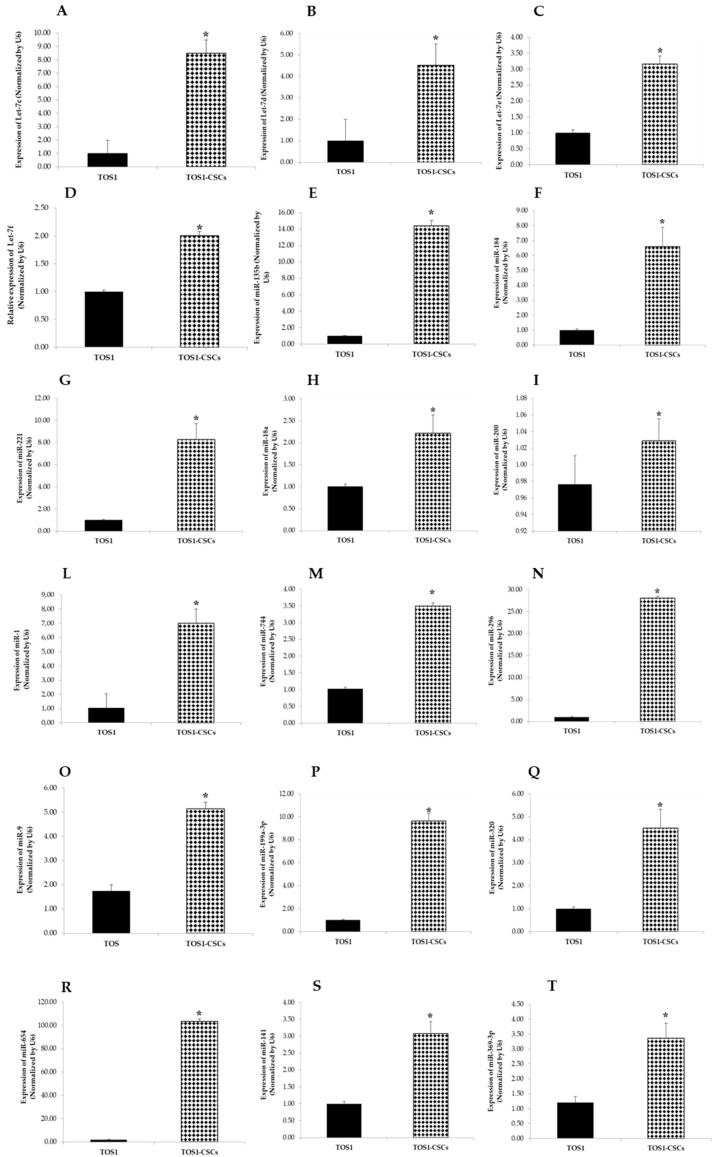
Expression levels of 18 miRNAs in TOS1-CSCs line. RT-qPCR analysis of expression of Let-7c (**A**), Let-7d (**B**), Let-7e (**C**), Let-7f (**D**), miR-135 (**E**), miR-184 (**F**), miR-221 (**G**), miR-18a (**H**), miR-200 (**I**), miR-1 (**L**), miR-744 (**M**), miR-296 (**N**), miR-9 (**O**), miR-199a-3p (**P**), miR-320 (**Q**), miR-654 (**R**), miR-141 (**S**), and miR-369-3p (**T**) in TOS1 and in TOS1-CSC cell lines. Data represent the mean with standard deviation (*n* = 4); * *p* < 0.001 as compared to TOS1 cell line.

The expression patterns of the 24 analyzed miRNAs (Let-7a, Let-7c, Let-7d, Let-7e, Let-7f, Let-7g, miR-25, miR-135b, miR-221, miR-423-5p, miR-365, miR-184, miR-9, miR-18a, miR-200b, miR-1, miR-744, miR-269, miR-500, miR-320, miR-654, miR-141, miR-199a-3p, and miR-369-3p) in the TOS1-CSC cell line was measured by RT-qPCR assay. Results showed that Let-7a, Let-7c, Let-7d, Let-7e, Let-7f, miR-9, miR-135b, miR-221, miR-184, miR-18a, miR-200, miR-1, miR-744, miR-296, miR-199a-3p, miR-320, miR-654, miR-141, and miR-369-3p were notably upregulated in the TOS1-CSCs line compared to the TOS1 cell line (Figure 12A–I,L–T).

**Figure 13:**
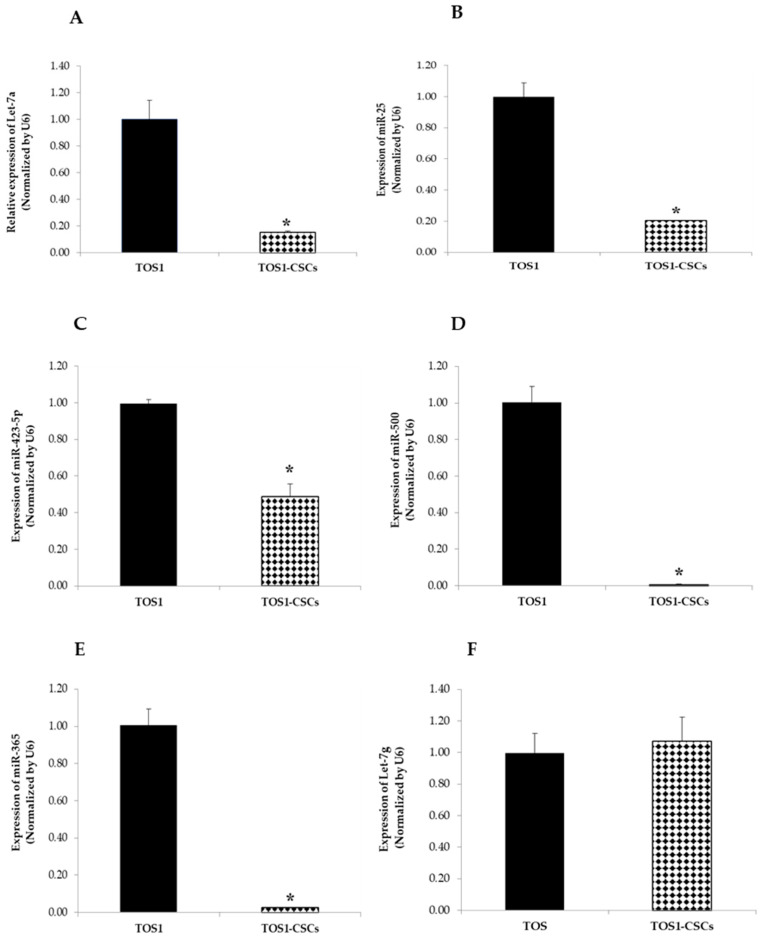
Expression levels of four miRNAs in the TOS1-CSC line. RT-qPCR analysis of expression of Let-7a (**A**), miR-25 (**B**), miR-423-5p (**C**), miR-500 (**D**), miR-365 (**E**) and Let-7g (**F**) were examined by real-time RT-PCR analysis in both TOS1 cell line and isolated TOS1-CSC cell line. Data represent the mean with standard deviation (*n* = 4); * *p* < 0.001 as compared to TOS1 cell line.

On the contrary Let-7a, miR-25, miR-423-5p, miR-365, miR-500, and miR-365 were notably downregulated compared to the TOS1 cell line. Results obtained showed also that there is no difference in the expression levels of Let-7g in the TOS1-CSCs cell line compared to the TOS1 cell line (Figure 13A–F).

In summary, this last showed a miRNA expression profile characteristic of the established TOS-CSCs in vitro model.

## 3. Discussion

Osteosarcoma (OS), the most common primary bone tumor, principally arising in the long bones of children and young adults, presents several high-grade subtypes, such as telangiectatic osteosarcoma (TOS) [1,2,6]. At the beginning of the 20th century, TOS was not recognized as a variant of OS, but was classified based on the histologic aspect as a hemorrhagic aneurism of bone [21,22]. In 1922, Ewing, on the grounds of further investigation, defined TOS as an aggressive variant of OS. TOS is a high-grade type of OS arising at the metaphyseal level of long bones, in particular, in the proximal tibia and distant femur [23]. This intraosseous tumor presents several characteristic histologic aspects, such as the presence of several cavities filled with clotted blood and segmented by several septa, in which it is possible find several types of tumor cells (i.e., endothelial, fibroblast and osteoblast-like cells), which, in addition to characteristic radiographical and prognostic aspects, show that TOS is a specific and quite different type of OS [24].

Another important aspect is that, as reported in several case reports, TOS can affect unusual skeletal sites (i.e., jaws, mandible, spine and skull) and soft tissues [12,13,14,15,16,17,18,19]. Recent findings have demonstrated that, although it seems that surgery and adjuvant/neoadjuvant chemotherapy could improve the survival rate of the patients who present TOS of the extremities, without metastases at the diagnosis, prognosis is still poor. In particular, for all the patients who receive a diagnosis of TOS in unusual sites or in soft tissues, the survival rate after surgical treatment, when possible, is poor, because recurrences or metastases in the lung are often present [12,13,14,15,16,17,18,19,27,28].

In recent decades, the emerging new concept of a particular tumor cellular subset constituted by Cancer Stem Cells (CSCs), which play an important role in both solid and liquid tumors, has paved the way to a new research approach regarding all tumors [35,36,37,38]. Currently, several research groups are investigating how CSCs are involved and which role they play in tumor progression, development, and metastasis, and if CSCs could be new targets for the development of new anticancer treatments. Several studies have shown the presence of CSCs in conventional OS [42,43,44]. Therefore, we have decided to investigate the possibility that TOS can also present CSC, as the mechanisms at the base of TOS progression and recurrence are still largely unknown. In the last year, we have had the opportunity to collect fresh bioptic samples of high-grade types of OS. From a collected rare bioptic sample of TOS, we have isolated a primary finite cell line, marked as TOS1. After that, and after confirmation by a pathologist of the tissue histotype, we proceeded to try to find CSCs in TOS. Following the experiments reported by Gibbs et al. [45] and by Palmini G. et al. [42] on the isolation of CSCs, using the ability of CSCs to grow and survive in stressful conditions, in this study, we isolated a putative CSCs line from the TOS1 primary cell line, marked as TOS1-CSCs line. It was observed that TOS1-CSCs at the second passage of subculture were able to self-renew, as demonstrated by the sarcosphere formation assay, which gave origin to a second generation of TOS-CSCs (data not shown). The differentiation experiments have confirmed the capacity of the isolated TOS1-CSCs line to differentiate in three mesodermal lineages of osteoblasts, adipocytes, and chondrocytes. In addition to this, the mesenchymal phenotype of the TOS1-CSCs cell line has been confirmed by cytofluorimetric assay and by immunohistochemical staining, which revealed the very positive expression of CD44, CD105, and CD90, which are recognized to be mesenchymal stem cell (MSC) markers. Immunohistochemical assay has also shown important data regarding the mesenchymal phenotype of this cell line, which is the total absence of CD45, the hematopoietic specific antigen. Finally, the MSC phenotype was also confirmed using the CFU assay, which reported a clonogenic rate potential of 33%, a value that is perfectly in line with healthy MSCs lines. Furthermore, our experiments have evaluated and confirmed their embryonic phenotype, which is another important characteristic of CSCs. Immunohistochemical staining has confirmed the expression of the transcription factors, which are related to this phenotype and are recognized to be embryonic stem cell (ESC) markers (Nanog, POU5F1, Sox2 and KLF4). Our gene expression analyses have shown not only their expression, but also the expression of another gene related to the embryonic phenotype (Lin-28A). In addition, our qualitative and quantitative gene expression analyses have shown a significant upregulation of all the ESC marker genes in the isolated TOS1-CSC cell line. All these factors have been recognized to constitute the transcriptional core at the base of the maintenance of the pluripotency of ESCs and, consequently, also of CSCs [46,47,48,49,50].

In this way, CSCs are able not only to maintain the primary tumor bulk, but are also responsible for recurrences and metastases, even after the multimodal therapeutic treatments.

In this study, we have also evaluated several other characteristics, which in recent years have been described as related to the CSC phenotype. One of these is the presence of Aldheide Dehydrogenase activity. Aldheide Dehydrogenase 1 family, A1 (ALDH1A1), is a detoxification enzyme, which has been reported to be responsible for chemoresistance in cancer [51]. Our ALDH activity assay has shown high levels of ALDH1A1 in our isolated TOS-CSCs line. This aspect is perfectly in line with finding these high levels only in malignancies, since CSCs also have the role of protecting the differentiated cells of the tumor bulk. The presence of other genes, which are linked to the neoplastic phenotype of CSCs (c-Myc, PROM1, EZR and AXL), has also been reported in our gene expression experiments. EZR and AXL genes encode for two proteins, which are involved in the invasion capacity of neoplastic cells [52,53]. In relation to this, we have also positively evaluated the invasion capacity of the isolated TOS1-CSCs line using the in vitro agar soft assay (data not shown), which is used to evaluate the capacity of tumor cells to invade other tissues.

After the characterization of the isolated cell line as a putative CSCs line, we decided to investigate what the expression signature profile of this unique cell line could be, since nothing similar has been done previously. In recent years, microRNAs (miRNAs), which are a group of small, single strand, non-coding RNAs able to post-transcriptionally regulate one or more target gene expression, and which have been described as important molecules involved in tumor development, progression and metastasis, have been reported to play an important role in bone sarcomas [33,34,54,55,56,57]. Several studies have begun to elucidate the role of miRNAs as oncogenes or tumor suppressors in OS. [56,57,58,59]. Despite the new findings, researchers are still far from clearly understanding the role of investigated miRNAs in the pathogenesis of OS. In addition, only a few research groups have investigated the presence and biology of CSCs in human high-grade types of OS [60,61], and even less have investigated the expression profile and the role of miRNAs in this particular cancerous cellular subset.

Hence, on the grounds of our previous findings, we hypothesized the presence, confirmed here, of CSCs in this aggressive variant of OS. After that, we carried out a preliminary evaluation of the miRNA expression profile in the established TOS-CSC in vitro model.

This study found that, in an analyzed panel of 24 miRNAs (Let-7a, Let-7c, Let-7d, Let-7 e, Let-7f, Let-7g, miR-25, miR-135b, miR-221, miR-423-5p, miR-184, miR-9, miR-18a, miR-200b, miR-1, miR-744, miR-296, miR-500, miR-320, miR-365, miR-654, miR-141, miR-199a-3p, and miR-369-3p), 18 resulted to be upregulated, one resulted with no difference in expression levels, and four resulted to be downregulated, as compared to the TOS1 cell line. This was the first time that several miRNAs were investigated not only in TOS but, especially, in an in vitro model of osteosarcoma cancer stem cells (OS-CSCs).

Of the group of 18 upregulated miRNAs (Let-7c, Let-7d, Let-7e, Let-7f, miR-135b, miR-221, miR-184, miR-18a-5p, miR-200, miR-1, miR-9, miR-744, miR-296-5p, miR-199a-3p, miR-320, miR-654, miR-141-3p and miR-369-3p), only miR-1, Let-7d, miR-320, miR-221, miR-184, miR-141-3p, miR-18a-5p, miR-135b, Let-7a, miR-199a-3p, miR-365, and miR-744 had been investigated [61,62,63,64,65,66,67,68,69,70,71,72,73,74,75,76,77,78,79,80,81,82,83] in OS before now.

Mir-1, described as a tumor suppressor in many types of cancer [84,85], has been reported to be downregulated also in OS cells, in which it directly inhibits the protein expression of VEGFA [63], with the consequent inhibition of cell proliferation, migration, and invasion.

Wu H. et al. described a lower expression of miR-320 human OS cell lines with respect to noncancerous tissue and human normal osteoblast cell lines, showing that the reintroduction of miR-320 in cancerous cells leads to an inhibition of cell proliferation by arresting the cell cycle in the G_1_ phase. They demonstrated that it is possible because miR-320 directly targets and activates the E2F1, a cell cycle regulator, by base pairing to its 3′-UTR [64].

Xu et al. and Wang J et al. have both reported that the expression of miR-141-3p in OS was low. Both the research groups have demonstrated that miR-141-3p is involved in the modulation and control of tumor growth and apoptosis [73,74].

Xu et al. reported that the presence of miR-141-3p can be related to an induction of apoptosis in human OS cell lines (MG63 and U2OS), hypothesizing that this effect is mediated by binding the binding sites of ZEB1 and ZEB2, which are two genes previously described as implicated in epithelial to mesenchymal transition (EMT) and tumor metastasis [86,87,88]. Wang J et al. described that a high expression of miR-141-3p, by targeting EGFR, can inhibit OS recurrence, leading to the hypothesis that high levels of miR-141-3p in OS patients could indicate a better prognosis than in patients with low expression levels.

Recent studies on the role of miR-199a-3p in several types of tumor have reported that downregulation of miRNA seems to be correlated to an increase in drug sensitivity [89,90]. These findings have also been reported by Gao Y. et al. and Lei W. et al., both of whom have investigated the role of miR-199a-3p.

Their studies have demonstrated that if miR-199a-3p, which has been found to be downregulated in OS tissue samples and cell lines, is overexpressed, there is an increase in OS chemosensitivity, by targeting CD44 or AK4 genes [79,80].

On the contrary to what has been described for the expression of miR-1, miR-320, miR-141-3p, and miR-199a-3p, our data have shown that these four miRNAs are upregulated in our TOS-CSC model. Therefore, we have hypothesized that these miRNAs can function in a different way, enhancing tumor progression, as has been described for other types of tumors [83,84,86,89,91].

In relation to miR-320 and miR-199a-3p, the high expression levels reported in our study could be related to enhanced drug sensitivity of human TOS by targeting, for example, FOXM1, as reported for colon adenocarcinoma by Wan L. Y et al. [92], and by binding the 3′-UTR region of CD44, as reported by Gao Y. et al. [79]. Our findings, in relation to both these miRNAs, could explain the partially good response to adjuvant/neoadjuvant chemotherapeutic treatments in patients affected by TOS of the extremities.

Therefore, while until now miR-320, miR-141-3p, miR-1, and miR-199a-3p have been reported to be low expressed in OS tissues and cells, miR-18a-5p, miR-135b, Let-7d, miR-184, miR-744, and miR-221 have been described as highly expressed in OS [61,65,71,72,75,76,83].

Lu C. et al. described that miR-18a-5p, which has been found to be involved in development, occurrence and clinical outcomes of several tumors [93,94,95], was upregulated in MG63 and SaOS-2. They reported that miR-18a-5p acts as an oncogene leading to OS cell proliferation and invasion, targeting and consequently inhibiting IRF2 [75], which has been reported to act as a functional regulator in cancers [96]. Consistent with this data on the role of miR-18a-5p in OS, recent studies have shown that miR-18a-5p functions in the same way, also targeting IRF2 in various tumors [97,98,99].

miR-135b, which has been reported to be involved in impaired osteogenic differentiation and in a negative regulation of osteogenesis from mesenchymal cells, was in previous years described as one of the miRNAs upregulated in OS and related to a poor prognosis, similar to other tumors. Pei H. et al. described that miR-135b is inversely related to FOXO1 pathways, showing that an upregulation of FOXO1, mediated by the inhibition of miR-135b, induces a progressive OS cell growth and invasion, and contemporary downregulation of miR-135b. Therefore, their study provided interesting evidence that miR-135b functions as an onco-miRNA in OS through negatively targeting FOXO1 [76].

miR-184 has been described to be closely related to tumor occurrence and progression in several tumors [97,98], among which OS. Du Z. et al., investigating the role of miR-184 in OS cell lines, have demonstrated that miR-184 is responsible for an abnormal cellular proliferation by upregulating Wnt and β-catenin mRNA expression levels [69]. Yin G. et al. reported that miR-184 is highly expressed in OS cell lines and demonstrated that the inhibition of this leads to a suppression of cell proliferation [72], confirming the oncogene behavior of this miRNA, as has been described in other malignant tumors [99].

One year later, Tao P. et al. confirmed that high expression levels of miR-184 are correlated to OS progression; in fact, the inhibition of miR-184 induces cellular apoptosis, reducing cellular proliferation and migration [71].

At the same time, Lin et al. reported in their in vitro study that treatment with doxorubicin induced a time-dependent expression of miR-184 which, targeting BCL2L1, induced a downregulation of this gene. BCL2L1 has been demonstrated to be involved in one of the molecular pathways that has been shown to play an important role in determining the effectiveness of adjuvant or neoadjuvant chemotherapeutic treatments in several tumors [100,101]. Data reported have effectively shown that the upregulation of miR-184 can lead to a poor therapeutic response by targeting BCL2L1 [70]. Among the miRNAs that we have analyzed in this study, until now, Let-7d is the only miRNA that has been investigated in a cellular model of OS-CSCs [61].

Di Fiore et al. have highlighted the double role that Let-7d can have in OS-CSCs. Initially, they found that the Let-7d was downregulated in their OS-CSCs model, demonstrating that a reintroduction of let-7d and its overexpression reduced tumorigenic and stemness properties of OS-CSCs. Moreover, they reported that Let-7d induced mesenchymal-epithelial transition, which was accompanied by an enhanced invasive capacity and reduced chemosensitivity in an OS-CSCs model [61].

Regarding miR-221, it has recently been observed that it can be involved in OS initiation, progression, and metastasis. miR-221 was recently found to be upregulated in OS cell lines with respect to normal human osteoblast cell lines, and this overexpression was positively correlated to OS cell proliferation, invasion, migration and cisplatin resistance [68]. Therefore, in this study by Yu W. et al. miR-221 was described to function as an oncomiR in OS. Similar findings have also been found by Hu X-H. et al. who described it as a downregulation of miR-221 suppressed OS progression [67]. Contemporarily, Gong N. et al. and Zhao H. et al. have both reported the importance that miR-221 can have as biomolecular marker for chemotherapeutic treatments [65,66].

Consistent with the above-mentioned studies on the upregulation of miR-18a-5p, miR-135b, Let-7d, miR-184 and miR-221 in OS cell lines and tissues, our results have shown that these five miRNAs are upregulated in our established TOS-CSC line. In relation to this, we have hypothesized that these miRNAs can function in TOS-CSCs similarly to that which has been reported in the studies on OS mentioned above. Future studies on their function in TOS-CSCs will be performed.

In this study, we also investigated the levels of expression of Let-7c, Let-7e, Let-7f, miR-200, miR-744, miR-296-5p, miR.9, miR-654, and miR-369-3p.

Our data show that all these miRNAs are upregulated in our TOS-CSC line. None of these miRNAs have been previously studied in OS cells and tissues or in CSCs, except for miR-744, which has been studied by Sun L. et al. [83] in OS cell lines.

The Let-7 family is known as one of the classic groups of miRNAs that play critical roles in carcinogenesis [102,103,104,105]. No data have been reported up to now on the functions of Let-7c, Let-7e and Let-7f in OS.

Previous studies have shown that miR-9 is regulated in several cancers and that miR-9 plays a crucial role in tumorigenesis and tumor progression, exercising different effects in different types of cancer [106,107]. In relation to the presence of this miRNA in OS, Xu S. et al. have found that miR-9 is upregulated in OS tissues compared to non-cancerous tissues, and that this overexpression can be correlated with the tumor size and the aggressive progression of OS [108]. Gang W. et al. have demonstrated that the inhibition of miR-9 enhances OS cell apoptosis and inhibits their metastatic potential [109].

Liu P. et al. have reported that miR-369-3p, similarly to miR-365, can also act as a tumor suppressor, inducing apoptosis and inhibiting cell migration by targeting ATG10 in endometrioid adenocarcinoma [110]. On the contrary, miR-200, miR-296-5p and miR-654 have been described in different studies as oncomirs [86,111,112,113,114]. Maia D. et al. have reported that the high expression levels of miR-296-5p mediate drug resistance and is correlated to tumor recurrence in early-stage laryngeal carcinoma [115]. Yoon A. et al. have demonstrated that miR-296-5p is enriched in cancer cells and, by downregulation of the p53-p21^WAF1^ pathway, contributes to tumorigenesis [109]. miR-654-5p has also recently been identified as an oncomir through Ras/MAPK signaling in oral squamous-cell carcinoma [111]. miR-744 has been described in several tumors as a miRNA correlated to a poor prognosis [116,117].

Sun et al. have reported that the overexpression of miR-744 in OS is most likely responsible for accelerating tumor growth and metastasis by targeting the LATS2 gene, which is able to control the cell cycle through arrest in the G_1_/S phase [83]. In the study of miR-744, we found high expression levels of this miRNA also in our TOS1-CSCs. Hence, we hypothesize that this could be another miRNA involved in the biology of CSCs and in their clonogenic ability.

In relation to these findings regarding the functions as tumor suppressors or oncomirs of these miRNAs, our future prospective is to investigate, according to previous studies in other cancers, their possible function in TOS-CSCs.

Finally, among the 24 miRNAs analyzed, our data have reported that Let-7a, miR-500, miR-365, miR-25 and Let-7a are all downregulated in the TOS-CSC line.

Iwasaki T. et al. have reported that Let-7a was significantly downregulated in all tested OS cells compared to mesenchymal stem cells, and that the E2F2 was significantly upregulated in OS cells compared to mesenchymal stem cells. Previous studies have reported that an abnormal expression of E2F2 can lead to an abnormal proliferation and to tumorigenesis. Hence, an overexpression of Let-7a, which, by negatively targeting E2F2 is responsible for inhibiting cell proliferation, can function as a tumor suppressor [77].

Zhang L. et al. and Zhang Z. et al. found, in gastric and prostate cancer, respectively, that miR-500 functions as an oncomir, which promotes, in both cases, cancer cell proliferation, survival and tumorigenicity through the activation of the NF-kB-signaling pathway [118,119] or through the inhibition of LRP1B.

miR-365 has been described as being involved in the tumorigenesis of various cancers [120,121]. Recently, Xu Y. et al. have shown that miR-365 was downregulated in OS cell lines and that reintroduction of this miRNA inside the OS cells induced an inhibition of OS proliferation by enhancing apoptosis [81].

The precise role of miR-25 in OS progression remains unclear. Chen B. et al. have demonstrated that miR-25 was significantly downregulated in OS cell lines and that an overexpression of this was correlated to the inhibition of epithelial mesenchymal transition and tumor progression by targeting SOX4. Therefore, miR-25 can be considered a tumor suppressor of OS. They also reported that levels of miR-25 were inversely correlated to levels of expression of SOX4. High levels of SOX4 recovered miR-25 induced tumor inhibition [122]. Recently, Yoshida A. et al., studying clinical and functional significance of intracellular and extracellular miR-25 in OS, have reported that miR-25 at intracellular level was implicated in chemotherapy resistance in OS, and that miR-25 inside the exosomes was responsible for inducing angiogenesis through the activation of endothelial cells, in this way promoting the metastatic process [123].

According to reported studies on miR-25, Let-7a, and miR-365 expression levels, our data confirmed a downregulation of these miRNAs in our established TOS-CSC line.

As for the other miRNAs investigated, further study of their function and the pathways influenced will be performed in the isolated cellular model of putative TOS-CSCs described here.

## 4. Materials and Methods

This study was conducted following approval of the Florence University Hospital Ethics Committee (Rif. N. 141/12), and has therefore been performed in accordance with the ethical standards laid down by the 1975 Declaration of Helsinki and its later amendments. Informed Consent for tissue collection, use, and storage of the samples was obtained from the donor at AOUC.

### 4.1. Primary Telangiectatic Osteosarcoma Cell Culture

Primary telangiectatic osteosarcoma (TOS) cell culture was produced in our laboratory from a fresh sample of TOS biopsy collected at the “Unità Ortopedia Oncologica e Ricostruttiva”, AOUC Careggi, Florence. The biopsy, obtained via needle aspiration, was immediately placed in a culture medium supplemented by 100 IU/mL penicillin and 100 μg/mL streptomycin, pH 7.4, and transported to the laboratory where it was processed.

The TOS primary cell culture, from the TOS tissue sample, was set up after enzymatic treatment in Ham’s F12 Coon’s modification medium (Sigma-Aldrich, St. Louis, MO, USA) with collagenase type II (Sigma-Aldrich, St. Louis, MO) at 37°, after mechanical dispersion. Cells were cultured as monolayer in growth medium (GM) Ham’s F12 Coon’s modification medium supplemented with 10% fetal bovine serum (FBS) in a modified atmosphere of 5% CO_2_ in air at 37 °C. GM was refreshed every three days. The TOS cell culture was signed as TOS1. When TOS1 cells grew to approximately 90% confluence, they were subcultured or harvested using Trypsin-EDTA.

### 4.2. Sarcosphere Formation Assay

This in vitro assay was used to identify and isolate cancer stem cells (CSCs) from the established TOS1 primary cell line. At 90% confluence in GM, monolayer cells were dissociated with Trypsin-EDTA into a single-cell suspension. The cells were inoculated into sarcosphere growth medium (SGM), supplemented 2% sterile methylcellulose (MC), at a density of 4 × 10^4^ cells/well in ultra-low attachment six-well plates (Corning Inc., Corning, NY, USA). SGM medium consists of 2× Ham’s F12 Coon’s modification medium supplemented with progesterone (20 nM), putrescine 100 µM, sodium selenite (30 nM), transferrin (25 µg/mL), insulin (20 µg/mL), human recombinant epidermal growth factor (EGF; 10 ng/mL), and basic fibroblast growth factor (b-FGF; 10 ng/mL), as described by Palmini G. et al. [42]. All reagents were purchased from Sigma-Aldrich. Fresh aliquots of b-FGF and EGF were added every three days.

After 21 days culture, spherical and floating colonies formed by >50 cells were defined “sarcospheres”, quantitated by inverted phase contrast microscope, and then isolated and plated in normal attachment 60 mm diameter tissue culture plates with normal GM. The cells isolated from sarcospheres were signed as telangiectatic osteosarcoma cancer stem cells (TOS1-CSCs). When TOS1-CSCs reached approximately 80 to 90% confluence, they were harvested and subcultured into a 100 mm diameter tissue culture plate for subsequent characterization analyses.

### 4.3. Telangiectatic Osteosarcoma Cancer Stem Cell Culture

TOS1-CSCs line was cultured in a specific growth medium (GM), which is composed of Ham’s F12 Coon’s modification medium supplemented with 10% FBS, 100 IU/mL penicillin, 100 μg/mL streptomycin, and 1 ng/mL b-FGF, to maintain their stemness profile. The medium was refreshed twice a week and the cells were used for cryopreservation and for in vitro analyses to characterize their stem-like phenotype upon reaching 5 × 10^3^ cells/cm^2^.

### 4.4. Cancer Stem Cell Phenotype Characterization

The characterization of the cancer stem cell phenotype of the isolated TOS1-CSCs line was performed by soft agar assay, colony forming unit (CFU) assay, osteogenic and adipogenesis differentiation assays, Aldehyde Dehydrogenase (ALDH) activity analysis, flow cytometric analyses, immunofluorescence staining, and by gene expression analyses.

#### 4.4.1. Soft Agar Assay

Soft Agar assay is an anchorage independent growth assay in soft agar, which is considered one of the most rigorous assays in vitro for detecting and proving the invasion capacity of cancerous cells. We performed soft agar assay on the primary cell line of TOS (TOS1), on the isolated TOS1-CSCs line, and on the human osteosarcoma cell lines Saos-2 purchased from American Type Culture (ATCC, Manassas, VA, USA), and on a mesenchymal stem cell line of preadipocytes (PA). A 35 mm dish was coated with 1% sterile agar prepared in culture medium maintained liquid at 47 °C.

The dish was immediately cooled. The cell lines in growth phase were detached, suspended in medium, diluted to double the required final concentration, and maintained at 37 °C. Then, 0.33% agar was prepared in medium and maintained at 45 °C. Cell suspension was mixed with an equal volume of 0.33% agar, distributed into the agar coated dish to obtain a final concentration of 2500 cells/dish, and immediately cooled. The cells were cultured at 37 °C in humidified air with 5% CO2 for 4 weeks until the formation of colonies and their growth. Colonies formed per dish were observed and counted in phase contrast microscopy (Axiovert 200, ZEISS). This experiment was performed in triplicate.

#### 4.4.2. Colony Forming Unit Assay

When TOS1-CSCs reached 80% confluence, they were detached with Trypsin-EDTA and plated in 100 mm diameter dishes with a final concentration of 450 cells/dish. The cells were cultured in Ham’s F12 Coon’s modification medium with 20% FBS, 100 IU/mL penicillin and 100 μg/mL streptomycin at 37 °C in humidified air with 5% CO_2_ for 4 weeks until the formation of colonies. Colonies formed per dish were stained with Toluidine Blue.

The colored colonies were counted using an inverted microscope (Axiovert 200, ZEISS). The CFU efficiency was calculated according to the following formula:

(Number of colonies formed/number of cells seeded) × 100.

This experiment was performed in triplicate.

#### 4.4.3. Adipogenesis Differentiation of TOS1-CSCs In Vitro

TOS1-CSCs line was cultured with a specific adipogenic medium (AM) in): in Ham’s F12 Coon’s modification medium supplemented with 10% (FBS), 100 IU/mL penicillin, 100 μg/mL streptomycin and 1 μM dexamethasone, 1 μM bovine insulin, and 0.5 mM isobutylmethylxanthine (IBMX). The medium was refreshed twice a week and the expression of the adipogenic phenotype was evaluated on cells cultured in AM or GM for 30 days by Oil Red O staining. The colored cells were observed in bright field microscopy (Axiovert 200, ZEISS).

#### 4.4.4. Osteogenic Differentiation of TOS1-CSCs In Vitro

TOS1-CSCs line was plated on 24-well plates at a cell density of 1 × 10^4^ cells/cm^2^ in GM and grown to 80 to 90% confluence in each well. Afterwards, the medium was switched to osteogenic medium (OM): Ham’s F12 Coon’s modification medium supplemented with 10% FBS, 100 IU/mL penicillin, 100 μg/mL streptomycin, 10 nM dexamethasone, 0.2 mM sodium L-ascorbyl-2-phosphate, and 10 mM β-glycerol phosphate. The medium was refreshed every three/four days. The osteogenic differentiation was stopped at 21 days to evaluate the osteoblastic phenotype. The cells were washed with DPBS (LONZA) (two times), fixed in 4% paraformaldehyde (PFA)/DPBS for 15 min, and washed with ultrapure water (three times). After that, for alkaline phosphatase (ALP) staining, the cells were washed with DPBS (two times) and stained with a specific dye mixture. This mixture is composed by Solution A (5 mg naphthol-AS-MX phosphate sodium salt dissolved in 1 mL dimethyl sulfoxide) and Solution B (40 mg Fast Blue BB dissolved in 49 mL Tris-HCl Buffer 280 mM, pH 9.0), which are mixed together forming Solution C. One mL of Solution C was added to each well for 30 min at 37 °C in humidified air with 5% CO_2_. ALP+ cells were stained in blue and nuclei were counterstained in red with Propidium Iodide. For mineralization staining, the cells were washed with DPBS (two times), fixed in 4% PFA/DPBS for 15 min, and washed with ultrapure water (three times). Calcium mineral deposits were stained for 2 min with 2% Alizarin Red S, pH 6.0, were rinsed with water and then stained in red-orange. ALP+ cells and calcium mineralized deposits were observed in bright field microscopy (Axiovert 200, ZEISS).

#### 4.4.5. Chondrogenic Differentiation of TOS1-CSCs In Vitro

To evaluate the chondrogenic potential of the isolated TOS1-CSC cell line, the cells were detached from culture flasks using 5 % Trypsin/EDTA and underwent chondrogenic differentiation in a 3D, high-density pellet culture using MesenCult™-ACF Chondrogenic Differentiation Medium kit (STEMCELL TECHNOLOGIES), following manufacturer’s instructions. After six days, fresh aliquots of complete MesenCult™-ACF Chondrogenic Differentiation Medium chondrogenic medium were added every three days. The same was also done on the TOS1 cell line.

Rounded formed pellets were harvested on day 21 for standard immunohistochemistry with Alcian Blue and for qualitative real time PCR (RT-PCR) for gene expression analysis of hyaline cartilage markers (Type X Alpha Collagen (COLXA1), Aggrecan (ACAN), Biglycan (BGN) and Decorin (DCN).

#### 4.4.6. Aldheyde Dehydrogenase Activity Assay

Aldehyde Dehydrogenase (ALDH) activity was evaluated by an ALDH activity colorimetric assay kit (Sigma-Aldrich, St. Louis, MO, USA) on the TOS1-CSCs and on a finite cell line of fibroblasts, used as negative control. This kit quantifies the ALDH enzymatic activity by absorbance reading at 450 nm (VICTOR3, Perkin Elmer). When the cell line reached 100% confluence, it was detached with Trypsin-EDTA. We then proceeded as described in the manufacturer’s protocol. All tests were done in triplicate.

#### 4.4.7. Immunofluorescence Staining

Immunofluorescence staining on TOS1-CSCs fixed in 4% PFA/DPBS was used to investigate the mesenchymal stem cell (MSC) markers, using primary antibody to CD44, CD45, CD90 and CD105, and to investigate the embryonic stem cells (ESCs) and the CSC markers, using, respectively, primary antibody to Nanog, POU5F1, SOX2, KLF4, Nestin and c-Kit. After fixation, cells were permeabilized by 0.2% Triton X-100/DPBS at 37 °C in humidified air with 5% CO_2_. Cells were washed three times with DPBS and were treated by RNase diluted 1/1000 with 2% BSA/DPBS at 37 °C in humidified air with 5% CO_2_. Cells were then washed three times in DPBS and stained with primary antibodies Anti-CD44 (Abcam) anti-CD45 (Abcam); anti-CD105 (Invitrogen, Carlsbad, CA, USA); anti-POU5F1 (Cell Signaling, Danvers, MA, USA); anti-PROM1 (Militeny), and with rabbit primary antibodies, anti-Nanog (Cell signaling); anti-SOX2 (Cell Signaling); anti-Nestin (Abcam); anti-KLF4 (Cell Signaling); anti-c-Kit (Bioss), anti-CD90 (Abcam). After incubation in a humid environment at 4 °C overnight, we removed the primary antibodies, and cells were stained with the secondary antibody (goat anti-Mouse Alexa Fluor 635 IgG (H + L), Life Technologies; Goat anti-Rabbit IgG (H + L) Superclonal Secondary Antibody, Alexa Fluor 488, Invitrogen), in the dark in a humid environment at room temperature for 45 min. Cells were then washed several times by DPBS and counterstained for nuclei with Propidium iodide (1:100 in DPBS). As negative internal control we used cells marked with only the secondary antibody. Stained cells were examined with 20× and 63× at room temperature on a Laser Scanning Confocal Microscopy (LSM 5109 Meta, ZEISS).

### 4.5. Gene Expression Analyses by Real-Time PCR

mRNA of TOS1, TOS1-CSCs, and of a finite primary cell line of human articular chondrocytes (ACs), was prepared using Trizol Reagent (Invitrogen, USA). Reverse transcription and Real Time PCR (RT-PCR) analysis were carried out as described using specific primers following the manufacturer’s protocol. β-Actin was used as internal control. First, we analyzed the expression of the SATB2 gene to analyze the phenotype of the primary cell line of TOS1. After that, we proceeded to characterize the CSC line. The expression of the ESCs and of the pluripotency marker genes (POU5F1, Nanog, SOX2, KLF4, LIN-28A) was evaluated on TOS1 and on TOS1-CSC lines. At the same time, on TOS1 and TOS1-CSC lines, the expression of the cancer stem cell marker genes (prominin 1 (PROM1) and MYC) and the expression of the marker genes for migration and metastasis (EZR and AXL) were also evaluated. All these analyses were set up on CSC cell lines cultured in GM at the third passage of subculture after the isolation of the sarcospheres and on the primary cell line cultured in GM at the first passage of subculture.

Finally, the expression of the adipogenic phenotype in the TOS1-CSCs was evaluated on cells cultured in GM or AM for 30 days by RT-PCR analysis of the marker genes peroxisome proliferator-activated receptor (PPARγ) and lipoprotein lipase (LPL). The expression of chondrogenic phenotype in TOS1-CSCs compared to a primary cell line of ACs (positive control of gene expression was evaluated on the spherical “pellet” after 21 days of chondrogenic differentiation of TOS1-CSC line, by RT-PCR analysis of the chondrogenesis gene markers (Aggrecan (ACAN), Biglycan (BGN), Decorin (DCN), and Type X Alpha 1 Collagen (COLXA1)).

The primer sequences used for amplification of all the genes described above are listed in Table 1. Moreover, the identity of each PCR product was resolved by 2% agarose gel electrophoresis stained with ethidium bromide.

### 4.6. Gene Expression Analyses by Quantitative Real-Time PCR

Total RNA of TOS1-CSC and TOS1 cell lines was extracted by using Qiazol Lysis Reagent (Invitrogen, USA). Reverse transcription and quantitative Real Time PCR (RT-qPCR) analyses were carried out as described using specific primers and probes (Table 2) which were designed by IDT integrated DNA technologies, following the manufacturer’s protocol. GAPDH was used as internal control. RT-qPCR was conducted using TaqMan Real-Time PCR Master Mix (Resnova, Roma, Italy) on a Rotor-Gene Q real-time PCR cycler (QIAGEN, Hilden, Germany). All points for standard curves and unknown samples were performed in triplicate. Student‘s *t*-test was used to determine the differences between TOS1-CSCs and TOS1. A *p*-value of <0.001 was considered statistically significant.

### 4.7. miRNA Analysis by RT-qPCR Assay

The expression levels of a panel of 24 miRNAs (Let-7a, Let-7c, Let-7d, Let-7e, Let-7f, Let-7g, miR-25, miR-135b, miR-221, miR-423-5p, miR-365, miR-184, miR-9, miR-18a, miR-1, miR-744, miR-269, miR-500, miR-320, miR-654, miR-141, miR-199a-3p, and miR-369-3p) in TOS1 and in TOS1-CSC cell lines were detected using a RT-qPCR assay.

Briefly, total miRNAs enriched RNA and after the total miRNAs fraction from TOS1 and TOS1-CSC lines was extracted with mirVana miRNA isolation Kit (Invitrogen) according to the manufacturer’s instructions. cDNAs for each miRNA were reverse transcribed from total miRNA fraction samples using specific miRNA primers from TaqMan Micro-RNA Assays (Applied Biosystems) and reagents from the TaqMan MicroRNA Reverse Transcription kit (Applied Biosystem, Foster City, CA, USA) according to the manufacturer’s instructions. Products were amplified by PCR using TaqMan Universal Master Mix II, with UNG (Applied Biosystems). Small nucleolar RNA U6 was used as the internal control standard for normalization. The cycle threshold (C_t_) was calculated. The 2^−ΔCT^ (ΔC_T_ = C_TmiRx_ − C_TU6 RNA_) method was used to quantify the relative amount of each analyzed miRNA. In addition, each measurement was performed in quadruplicate.

The statistical analysis was carried out by the Relative Expression Software Tool V 2.0.13 (REST 2009; Qiagen). Bonferroni‘s test was used to determine the differences between TOS1-CSCs and TOS1 lines. A *p*-value of < 0.001 was considered statistically significant.

## 5. Conclusions

In this study, we have collected and established a human primary cell line of TOS. In addition to this, we have provided the evidence of the existence of CSCs in TOS. For the first time, we have established and characterized a putative CSCs line of TOS, providing their characteristic and specific in vitro features. We have established an in vitro cellular model which could be useful for understanding the biology of the most important cellular subpopulation of this rare and aggressive type of OS.

In relation to this, we have analyzed the expression profile of 24 miRNAs, which in part had never been investigated in OS cells and tissues, or in other types of cancers. They are now starting to be studied in high grade conventional OS with the final aim of improving knowledge on TOS tumorigenesis and progression. Therefore, we have presented the first and preliminary expression profile of miRNAs in a rare in vitro model of TOS primary cell line and of putative TOS-CSCs lines.

Further studies are needed to clarify and understand the effects of the miRNAs analyzed on TOS-CSC proliferation, invasion potential, and drug resistance, and whether there may be a synergic relationship. The final aims are to find new molecular targets for the development of more effective and non-invasive therapies against TOS, and to find new prognostic and diagnostic biomolecular markers which, in the future, will permit simple diagnosis and evaluation of response to treatment in patients affected by TOS.

## Figures and Tables

**Table 1:** Detailed list of primer sequences, with the amplicon size and annealing temperature.

Gene	Oligonucleotides	Sequence (5′-3′)	Amplicon Size	T_a_ (°C)
*SATB2*	Forward Reverse	TGTCTATCATGTTGTGACGTTGA TCATCTCTTTGAGCAGTTCCTTTA	150 bp	63
*Nanog*	Forward Reverse	CCCAGCTGTGTGTACTCAAT GGTTCAGGATGTTGGAGAGTT	87 bp	60
*POU5F*1	Forward Reverse	GGGAGAGCTAGGGAAAGA TCCTTCCTTAGTGAATGAAGAACT	77 bp	60
*Sox2*	Forward Reverse	TGCAGTACAACTCCATGA GGACTTGACCACCGAACC	125 bp	55
*KLF4*	Forward Reverse	CGGGAAGGGAGAAGACACT AGTCGCTTCATGTGGGAGA	79 bp	60
*LIN28A*	Forward Reverse	CGACTGTAAGTGGTTCAAC CCTTCCATGTGCAGCTTACT	100 bp	60
*MYC*	Forward Reverse	GCTGCTTAGACGCTGGATTTTT GAGTCGTAGTCGAGGCATAGT	110 bp	63
*PROM1*	Forward Reverse	CCAGAAGCCGGGTCAAAAT ATTCACTCAAGGCACCATCC	127 bp	60
*EZR*	Forward Reverse	GCCTTCTTGTCGATGGGTTA GCCTCTTGTCGATGGGTTTA	134 bp	61
*AXL*	Forward Reverse	TTAGTGCTACGCGGAATGG CCTATGTCCATAGCACCTCG	133 bp	60
*PPARγ*	Forward Reverse	GTCGGTTTCAGAAATGCCTTG ATCTCCGCCAACAGCTTC	97 bp	57
*LPL*	Forward Reverse	TGCATTTCAATCACAGCAGCAA TACAGGGCGGCCACAAG	101 bp	57
*COLXA1*	Forward Reverse	AGAGGTGAAAATGGGGTTCCA GGCAAGCCTGGTTTCCCAAA	248 bp	60
*ACAN*	Forward Reverse	GGGTCAACAGTGCCTATCAG GGGTGTAGCGTGTAGAGATG	213 bp	62
*BGN*	Forward Reverse	ACCTCCCTGAGACCCTGAAT CACCCACTTTGGTGATGTTG	273 bp	62
*DCN*	Forward Reverse	CACCAAAGTGCGAAAAGTTAC CTTAGCCAAATTATTCAGTCCTT	261 bp	60
*β-actin*	Forward Reverse	AGCCTCGCCTTTGCCGA CTGGTGCCTGGGGCG	174 bp	60

bp, base pairs of amplicon size; T_a_ annealing temperature.

**Table 2:** Detailed list of primer and probe sequences, with the amplicon size and annealing temperature.

Gene	Primer Sequences and TaqMan Probes 5′-3′	Amplicon Size	T_a_ (°C)
*Nanog*	For. CCCAGCTTGTGTGTACTCAAT Probe. FAM/AATACCTCA/ZEN/GCCTCCAGCAGATGC/3IABkFQ Rev. GGTTCAGGATGTTGGAGAGTT	87 bp	60
*POU5F1*	For. GGGAGAGCTAGGGAAAGA Probe. FAM/AACCTGGAG/ZEN/TTTGTGCCAGG/3IABkFQ Rev. TCCTTCCTTAGTGAATGAAGAACT	77 bp	60
*KLF4*	For. CGGGAAGGGAGAAGACACT Probe. FAM/AATAACCGC/ZEN/TGGCGGGAGGA/3IABkFQ Rev. AGTCGCTTCATGTGGGAGA	79 bp	60
*SOX2*	For. TGCAGTACAACTCCATGA Probe. FAM/ACAGCATGT/ZEN/CCTACTCGCAGCAG/3IABkFQ Rev. GGACTTGACCACCGAACC	125 bp	60
*LIN28A*	For. GACTGTAAGTGGTTACAC Probe. FAM/TCATGGACA/ZEN/GGAAGCCGAACCC/3IABkFQ Rev. CGACTGTAAGTGGTTCAAC	104 bp	60
*GAPDH*	For. AATCCGTTGACTCCGACCTTC Probe. FAM/CCACATCGC/ZEN/TCAGACACCATGGG/3iaKfq Rev. ACAGTACAGCCGCATCTTC	179 bp	69

bp, base pairs of amplicon size; T_a_ annealing temperature.

## Data Availability

The data presented in this study are available on request from the corresponding author.

## Abbreviations

TOS	Telangiectatic Osteosarcoma
OS	Osteosarcoma
CSCs	Cancer Stem Cells
TOS-CSCs	Telangiectatic Osteosarcoma Cancer Stem Cells
miRNA	microRNA
GM	Growth Medium
LPL	Lipoprotein Lipase
PPARγ	Peroxisome Proliferator-Activated Receptor gamma
ALP	Alkaline Phosphatase
HA	Hydroxyapatite
COLXA1	Type X Alpha Collagen I
ACAN	Aggrecan
DCN	Decorin
BGN	Biglycan
COLXA1	Type X Alpha 1 Collagen
ACs	Articular Chondrocytes
MSCs	Mesenchymal Stem Cells
LSCM	Laser Scanning Confocal Microscopy
ESCs	Embryonic Stem Cells
CFU	Colony Forming Unit
ALDH1A1	Aldheide Dehydrogenase 1 family, A1
EMT	Epithelial Mesenchymal Transition
OM	Osteogenic Medium
Nanog	Homeobox protein NANOG
SOX2	(Sex determining region Y)-box 2
POU5F1	POU domain, class 5, transcription factor 1
KLF4	Kruppel Like Factor 4
Lin28A	Lin-28 homolog A
AXL	Tyrosine-protein kinase receptor UFO
EZR	Ezrin
MYC	MYC Proto-Oncogene
PROM1	Prominin-1
PPARγ	Peroxisome Proliferator Activated Receptor gamma
LPL	Lipoprotein Lipase
